# Early Hints of Metagenome Next‐Generation Sequencing and Copy Number Variations Analysis: An Occult Case of Leptomeningeal Metastasis With Rapid Cognitive Decline

**DOI:** 10.1002/ccr3.9676

**Published:** 2024-12-02

**Authors:** Xueqin Chen, Haotao Zheng, Taoli Wang, Ziyang Feng, Jia Wang, Yangsicheng Liu, Wenxin Qin, Fanxin Kong, Xiude Qin

**Affiliations:** ^1^ The Forth Clinical Medical College of Guangzhou University of Chinese Medicine Shenzhen Guangdong China; ^2^ Shenzhen Traditional Chinese Medicine Hospital Shenzhen Guangdong China

**Keywords:** case report, copy number variations, leptomeningeal metastasis, metagenome next‐generation sequencing

## Abstract

Early detection and treatment are critical for improving the prognosis of patients with leptomeningeal metastasis. However, heterogeneous clinical manifestations, non‐specific imaging, and limited sensitivity of cerebrospinal fluid cytology posed challenges to identifying leptomeningeal metastasis in the early stage. Here we reported a case of lung adenocarcinoma complaining of rapid cognitive decline, whose magnetic resonance imaging showed interstitial brain edema. Under the circumstances of negative cerebrospinal fluid cytology, metagenome next‐generation sequencing and copy number variations analysis were applied, which indicated leptomeningeal metastasis and was confirmed in the subsequent cytology.

**Summary**

- We reported a case of lung adenocarcinoma complaining of rapid cognitive decline, whose imaging showed interstitial brain edema.
- Under the circumstances of negative cerebrospinal fluid cytology, metagenome next‐generation sequencing and copy number variations analysis were applied, which indicated leptomeningeal metastasis and was confirmed in the subsequent cytology.

## 1 Introduction

Leptomeningeal metastasis (LM), as a devastating complication of metastatic cancer, is characterized by the dissemination of tumor cells throughout the subarachnoid space and leptomeninges [1]. Up to 10% of individuals diagnosed with positive epidermal growth factor receptor (EGFR) non‐small cell lung cancer may experience LM, whose median overall survival is only 4–6 weeks if untreated after diagnosis [2]. Early identification and intervention are critical for improving the prognosis of LM. Cerebrospinal fluid (CSF) cytology of tumor cells is still the golden standard for diagnosing LM with higher specificity compared with the hints of neuroimaging and neurological symptoms. However, early diagnosis of LM remains challenging given the limited sensitivity of CSF cytology that may require multiple samples and repeat lumbar punctures, as well as heterogeneous signs and symptoms at presentation. Therefore, exploring more sensitive diagnostic technologies is a potential research direction for LM.

Here, we introduced the integration of metagenome next‐generation sequencing (mNGS) and copy number variations (CNVs) analysis as a diagnostic tool for early‐stage LM.

## 2 Case History/Examination

A 54‐year‐old male was referred to the encephalopathy department from the oncology department due to his complaint of rapid cognitive decline. The patient was first diagnosed with lung adenocarcinoma with EGFR L858R mutation in September 2020, with metastasis to the pleura, contralateral lung, and bones. He was initially treated with osimertinib, a third‐generation EGFR tyrosine kinase inhibitor (TKI), which produced a positive biochemical response and clinical improvement. Gradually, with resistance to TKIs and disease progression according to the evaluation of positron emission tomography/computed tomography (PET/CT) scan results, several therapies were adjusted (Data S1).

**FIGURE 1:**
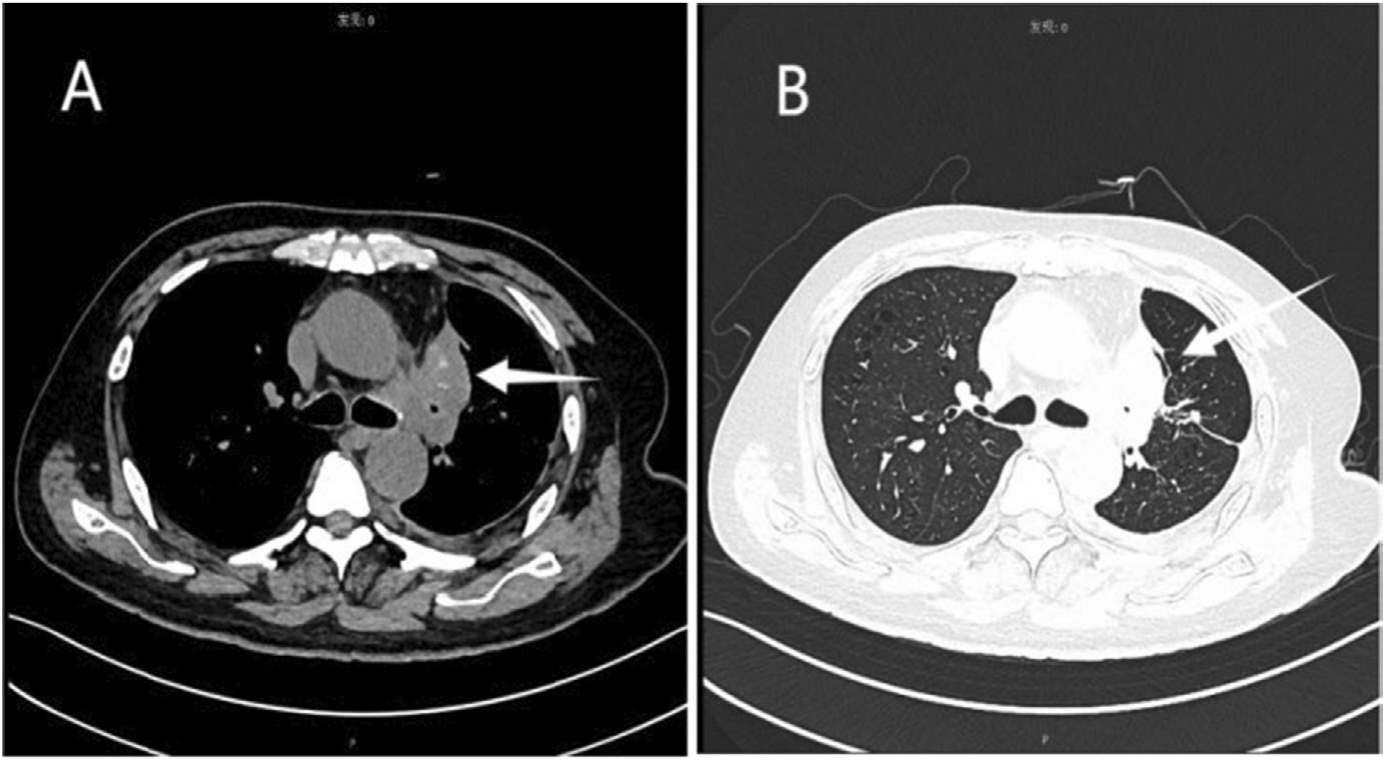
Chest scans of mediastinal window (A) and lung window (B). A lobulated massy shadow measuring 51 × 43 × 48 mm of uneven density with patchy calcification and burr edges was observed in the upper lobe of the left lung. The left pleura is thickened and stretched, along with the presence of a pleural effusion on the same side.

Most recently, 1 week after receiving bevacizumab and pemetrexed disodium, the patient developed asthenia and dizziness and was admitted to the oncology department after the symptoms persisted for a week. Following admission, contrast‐enhanced CT of the chest was assessed as continued stable disease (Figure 1). Besides, electromyography revealed peripheral nerve injury, especially in his lower limbs, which was considered to be related to the previous chemotherapy. However, abrupt rapid cognitive decline prompted the patient's referral to the encephalopathy unit on 2 August 2023.

**FIGURE 2:**
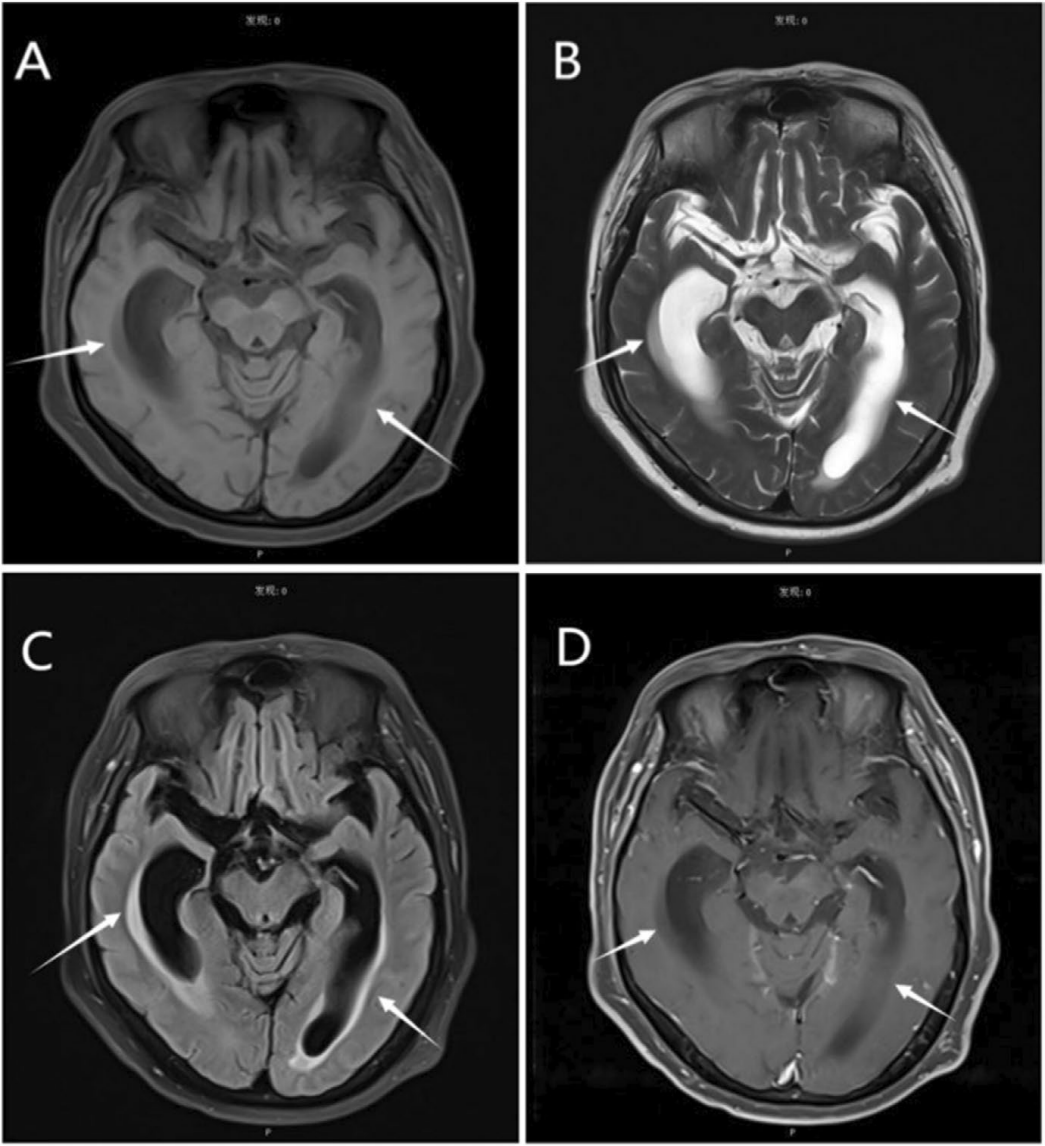
(A) T1‐weighted images (T1WI) showed mild dilatation of the ventricular system of the brain and decreased signal changes in the surrounding cerebral white matter. (B) T2‐weighted imaging (T2WI) represented increased signal. Fluid attenuation inversion recovery (FLAIR) manifested patchy enhancement near the bilateral Lateral ventricle (C), while no relative enhancement was found (D).

On the initial exam, the patient could not recognize his family. The mini‐mental state examination (MMSE) was 13. Unsteady gait and dysmetria on the finger‐to‐nose test were observed. Except for positive Romberg tests, his cranial nerve examination was intact, as were his strength, sensation, meningeal irritation signs, and reflexes throughout his extremities. Furthermore, enhanced cranial magnetic resonance imaging (MRI) revealed interstitial brain edema (Figure 2). CSF was also examined and a pressure of 220 mm H_2_O was recorded. CSF analysis showed normal protein (196.0 mg/dL), decreased glucose (2.70 mmol/L), and lactate dehydrogenase (4 U/L). Additionally, CSF culture, smears for *Cryptococcus neoformans* and acid‐fast bacilli, immunoglobulin M for Toxoplasma, rubella, cytomegalovirus, herpes simplex virus, and syphilis were all negative. CSF cytology revealed only a few lymphocytes.

## 3 Differential Diagnosis, Investigations, and Treatment

**FIGURE 3:**
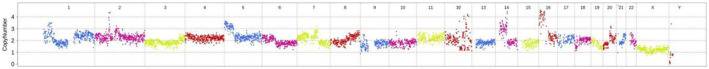
Image of copy number variation of CSF from Agene (Fuzhou) Genetic Medical Testing Laboratory. The numbers on the horizontal axis of the table represent the chromosome sequence and the vertical axis represents relative copy numbers. Chr1, Chr5, Chr9, Chr12, Chr14, Chr16, Chr18 and Chr20 were observed chromosomal abnormality.

**FIGURE 4:**
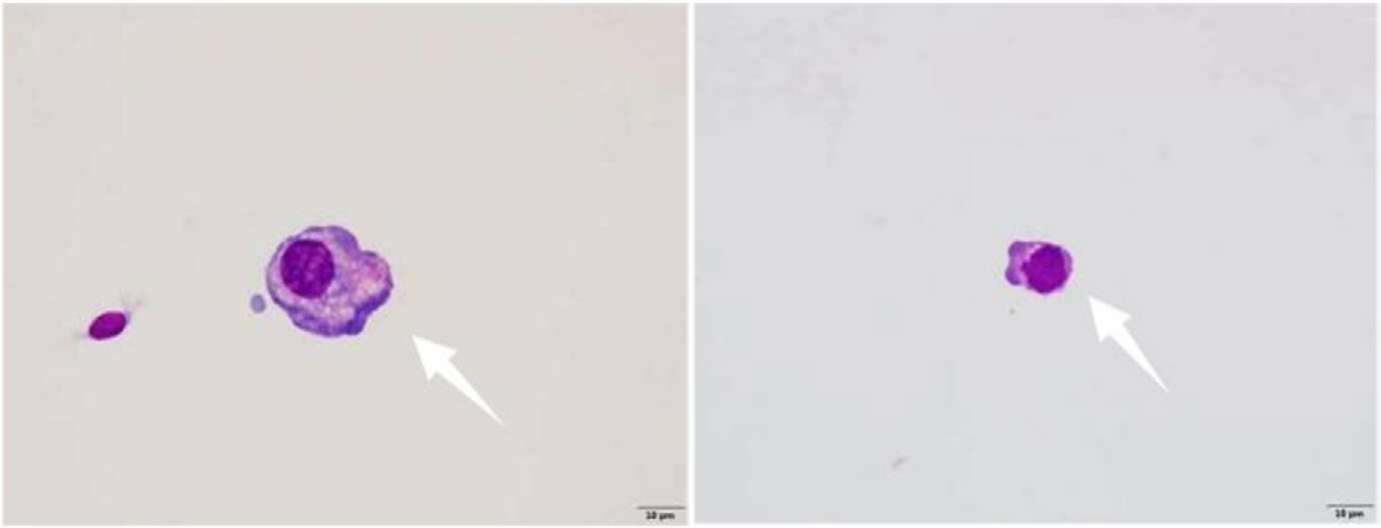
Tumor cells of CSF were irregular, with abundant cytoplasm and uneven staining. Besides, the nuclei are large, deviated, and deeply stained, and the nucleoli and nuclear membranes are relatively clear.

Considering the rapid cognitive decline, brain edema, and elevated CSF pressure, differential diagnoses included LM, cerebral ischemia, and meningitis caused by bacterial, viral, or fungal infections. Mannitol and hypertonic saline therapies were applied to release brain edema and reduce intracranial pressure, but little efficacy was achieved. More examinations were conducted to figure out the etiology. On 8 August, CNVs of CSF discovered nine chromosomal abnormalities (Figure 3), which indicated central nervous system (CNS) tumors. Besides, one sequence each of Cytomegalovirus and Epstein–Barr virus were found by mNGS of CSF. Based on the indications of mNGS and CNVs analysis, a repeat cytological examination of CSF was conducted, where tumor cells were discovered (Figure 4). The patient was eventually diagnosed with LM and referred to the oncology department for intravenous bevacizumab (15 mg/kg, every 21 days) and osimertinib (160 mg/day), since he could not withstand the potential side effects of intrathecal therapy [3].

## 4 Outcome and Follow‐Up

When discharged from the hospital, the patient's cognitive function improved significantly, but other neurological examinations did not reveal obvious improvement, probably due to the blood–brain barrier reducing bevacizumab and osimertinib's concentration.

## 5 Discussion

Parenchymal brain metastases and leptomeningeal metastases are the most prevalent adult intracranial malignancies, with increasing incidence on account of innovation and advancement in early identification. LM may occur in various advanced cancer patients, most of which arise from breast carcinoma, lung cancer, and lymphoma [4]. Due to the restrictions created by the blood–brain barrier, both cancerous cells and most therapeutic agents are prevented from accessing the CNS. Besides, significantly hypoxic CSF with low concentrations of micronutrients obstructs tumor cells from traveling through the leptomeningeal [5]. Despite the harsh microenvironments in CSF, aggressive tumor cells survive by competing for iron with macrophages [6], and spread to leptomeningeal via direct extension from brain metastases, hematogenous or lymphatic dispersion, as well as endoneural and perineural diffusion [7].

The diagnosis of LM is based on comprehensive assessments of clinical manifestations, imaging, and CSF cytology. Neurological symptoms at the time of brain metastasis diagnosis are a strong, independent predictor of survival time [8]. However, patients with LM have diverse symptoms that reflect different sites invaded by the tumor cells, including neuroaxial involvement, cerebral, cranial nerve, and spinal complaints. Heterogeneous clinical manifestations of LM pose challenges to clinicians, such as headache, ensuing nausea and vomiting, weakness, rapid weight loss, mental obtundation, excessive sleepiness, and speech disorders [9]. Notably, the early signs of LM can be subtle, and difficult to distinguish from the toxic effects of radiotherapy or chemotherapy used to treat the primary tumor. Therefore, clinicians should be vigilant when receiving patients with suspected LM and conduct careful neurological assessments.

Gadolinium‐enhanced MRI, sensitive to minor lesions, edema, and meningeal enhancement, is the best imaging technique to evaluate LM. Characteristic MRI findings that suggest LM are the enhancement of leptomeningeal nodules or cranial nerve roots, sulcal and folial obliteration or enhancement, and linear enhancement of the ependyma [10]. Nonetheless, a normal or false‐negative MRI is present in 20%–30% of patients diagnosed with LM [11]. Besides, an MRI should be performed before any irritation to the leptomeninges such as lumbar punctures or neurosurgery, which could induce false‐positive enhancement.

Visualization of malignant cells, decreased glucose concentration, increased protein, and lymphocytes are typical CSF findings for LM. However, up to 45% of LM patients tested negative in CSF cytology on the first examination [12]. The sensitivity of CSF cytology results has been demonstrated to increase by withdrawing more than 10.5 mL CSF, processing specimens immediately, obtaining CSF from the most optimal location, and repeating lumbar punctures [13]. Nevertheless, repeated punctures were usually required, which might result in local bleeding or infection, CSF leakage, and hypotensive cranial pressure headache. Our patient's clinical presentations were unique since neither rapid cognitive decline nor interstitial brain edema on imaging are typical of LM. Therefore, LM was not considered at first, especially when the previous CSF cytology was negative. Mannitol and hypertonic haline therapies were applied, aiming to improve the patient's symptoms, but with little efficacy. MNGS‐CNVs brought a turn to our treatment dilemma.

CNVs, as important contributors to genomic alterations, are structurally variant regions characterized by differences in copy numbers across two or more genomes, which confers substantial susceptibility to numerous forms of cancer [14, 15]. DNA segments longer than 50 base pairs can be deleted or duplicated in CNVs, and the number of copies of a given DNA segment can differ from that of the reference genome [16]. Seminal papers have demonstrated that CNVs can be used to predict the presence of tumor cells, or tumor progression, as well as treatment response [17, 18, 19]. Peripheral blood is a common detection source for CNVs, but LM lacks detectable CNVs in plasma samples owing to the blood–brain barrier. Instead, CNV detection in CSF is more sensitive and reliable for LM than plasma [20]. MNGS detects potential pathogens by obtaining genomic information of the sample species through nucleic acid fragments. In the absence of microbiological culture, mNGS is featured by unbiased sampling, which performs parallel sequencing of a large number of DNA sequences, broadly identifies known and unexpected pathogens, and even facilitates the discovery of new organisms [21]. Currently, appreciation of the role of mNGS in CSF in diagnosing neurological infections has burgeoned [22, 23, 24]. The combination of mNGS and CNVs suggested CNS malignancy in the presence of negative CSF flow cytometry or cytology with a sensitivity of 75% (95% CI, 63%–85%) and a specificity of 100% (95% CI, 96%–100%) [25].

In our case, given the patient's unexplained cognitive impairment and the possibility of false‐negative CSF cytology, mNGS‐CNVs were applied to analyze CSF for information on pathogenic microorganisms and tumor cell chromosomes' CNVs. As a result, nine variations above 5 Mb were detected in copy number, which indicated chromosome instability, in accordance with the chromosomal features of malignancy. Besides, mNGS detected no high‐sequence microorganisms, providing evidence to rule out infectious meningitis. Furthermore, considering the patient's history of lung cancer, LM was highly suspected, and repeat CSF cytology was performed. Eventually, tumor cells were discovered in the later cytology, confirming the diagnosis of LM and facilitating subsequent treatment. In addition, mNGS‐CNVs eliminate the need for preserving cell integrity with lower specimen volume requirements, simultaneously collecting more biological information and providing more diagnostic references for patients to distinguish neuroinfectious diseases and neurotumours with one lumbar puncture. Nonetheless, potential false positives or negatives of mNGS‐CNVs may necessitate cytological validation [25], and the higher cost and technology constraints limit its extensive implementation. Large clinical trials on the diagnosis of LM using mNGS‐CNVs are still lacking, and more relevant research will be encouraged in the future.

Early screening, identification, and diagnosis were crucial to initiating antitumor therapy and improving the survival rate of LM, especially for cancer patients with a sudden onset of unexplained neurological symptoms. Clinicians should attach importance to comprehensive evaluation, including clinical manifestations, imaging, and CSF cytology, in the management of ML. As a newly developed technology, mNGS‐CNVs may play a significant role in distinguishing LM in the early cancer stage. We recommend mNGS‐CNVs for patients with a high suspicion of LM who can undergo lumbar puncture. Future studies will be required to determine the efficacy and accuracy of mNGS‐CNVs in the diagnosis of LM.

## Author Contributions

**Xueqin Chen:** conceptualization, writing – original draft, writing – review and editing. **Haotao Zheng:** resources. **Taoli Wang:** resources. **Ziyang Feng:** data curation, formal analysis. **Jia Wang:** data curation, formal analysis. **Yangsicheng Liu:** data curation, formal analysis. **Wenxin Qin:** data curation, formal analysis. **Fanxin Kong:** conceptualization, writing – review and editing. **Xiude Qin:** funding acquisition.

## Consent

The patient provided written informed consent for the publication of this case report and the use of related pictures.

## Supporting information


Data S1.

## Data Availability

The data that support the findings of this study are available from the corresponding author upon reasonable request.

## References

[1] E. Le Rhun , M. Weller , D. Brandsma , et al., “EANO‐ESMO Clinical Practice Guidelines for Diagnosis, Treatment and Follow‐Up of Patients With Leptomeningeal Metastasis From Solid Tumours,” Annals of Oncology 28 (2017): iv84–iv99. 28881917 10.1093/annonc/mdx221

[2] S. A. Grossman and M. J. Krabak , “Leptomeningeal Carcinomatosis,” Cancer Treatment Reviews 25, no. 2 (1999): 103–119. 10395835 10.1053/ctrv.1999.0119

[3] D. M. Byrnes , F. Vargas , C. Dermarkarian , et al., “Complications of Intrathecal Chemotherapy in Adults: Single‐Institution Experience in 109 Consecutive Patients,” Journal of Oncology 2019 (2019): 4047617. 31186634 10.1155/2019/4047617 PMC6521528

[4] J. L. Clarke , H. R. Perez , L. M. Jacks , K. S. Panageas , and L. M. Deangelis , “Leptomeningeal Metastases in the MRI Era,” Neurology 74, no. 18 (2010): 1449–1454. 20439847 10.1212/WNL.0b013e3181dc1a69 PMC2871005

[5] Q. Li , Z. Lin , Y. Hong , et al., “Brain Parenchymal and Leptomeningeal Metastasis in Non‐Small Cell Lung Cancer,” Scientific Reports‐UK 12, no. 1 (2022): 22372. 10.1038/s41598-022-26131-z PMC9792549 36572759

[6] Y. Chi , J. Remsik , V. Kiseliovas , et al., “Cancer Cells Deploy Lipocalin‐2 to Collect Limiting Iron in Leptomeningeal Metastasis,” Science 369, no. 6501 (2020): 276–282. 32675368 10.1126/science.aaz2193 PMC7816199

[7] H. Cheng and R. Perez‐Soler , “Leptomeningeal Metastases in Non‐Small‐Cell Lung Cancer,” Lancet Oncology 19, no. 1 (2018): e43–e55. 29304362 10.1016/S1470-2045(17)30689-7

[8] A. Steindl , S. Yadavalli , K. A. Gruber , et al., “Neurological Symptom Burden Impacts Survival Prognosis in Patients With Newly Diagnosed Non‐Small Cell Lung Cancer Brain Metastases,” Cancer‐American Cancer Society 126, no. 19 (2020): 4341–4352. 10.1002/cncr.33085 PMC7540353 32678971

[9] Z. Pan , G. Yang , H. He , et al., “Leptomeningeal Metastasis From Solid Tumors: Clinical Features and Its Diagnostic Implication,” Scientific Reports‐UK 8, no. 1 (2018): 10445. 10.1038/s41598-018-28662-w PMC6041294 29992998

[10] E. Le Rhun , P. Devos , J. Weller , et al., “Prognostic Validation and Clinical Implications of the EANO ESMO Classification of Leptomeningeal Metastasis From Solid Tumors,” Neuro‐Oncology 23, no. 7 (2021): 1100–1112. 33367859 10.1093/neuonc/noaa298 PMC8301235

[11] J. W. Hyun , I. H. Jeong , A. Joung , H. J. Cho , S. H. Kim , and H. J. Kim , “Leptomeningeal Metastasis: Clinical Experience of 519 Cases,” European Journal of Cancer 56 (2016): 107–114. 26841095 10.1016/j.ejca.2015.12.021

[12] E. Le Rhun , F. Massin , Q. Tu , J. Bonneterre , M. C. Bittencourt , and G. C. Faure , “Development of a New Method for Identification and Quantification in Cerebrospinal Fluid of Malignant Cells From Breast Carcinoma Leptomeningeal Metastasis,” BMC Clinical Pathology 12 (2012): 21. 23145812 10.1186/1472-6890-12-21 PMC3539901

[13] M. J. Glantz , B. F. Cole , L. K. Glantz , et al., “Cerebrospinal Fluid Cytology in Patients With Cancer: Minimizing False‐Negative Results,” Cancer‐American Cancer Society 82, no. 4 (1998): 733–739. 10.1002/(sici)1097-0142(19980215)82:4<733::aid-cncr17>3.0.co;2-z 9477107

[14] A. Shlien and D. Malkin , “Copy Number Variations and Cancer,” Genome Medicine 1, no. 6 (2009): 62. 19566914 10.1186/gm62 PMC2703871

[15] P. H. Dear , “Copy‐Number Variation: The End of the Human Genome?,” Trends in Biotechnology 27, no. 8 (2009): 448–454. 19576644 10.1016/j.tibtech.2009.05.003

[16] M. Zarrei , J. R. MacDonald , D. Merico , and S. W. Scherer , “A Copy Number Variation Map of the Human Genome,” Nature Reviews. Genetics 16, no. 3 (2015): 172–183. 10.1038/nrg3871 25645873

[17] A. M. Bowcock , “Invited Review DNA Copy Number Changes as Diagnostic Tools for Lung Cancer,” Thorax 69, no. 5 (2014): 495–496. 24188925 10.1136/thoraxjnl-2013-204681

[18] R. A. van Boerdonk , J. M. Daniels , P. J. Snijders , et al., “DNA Copy Number Aberrations in Endobronchial Lesions: A Validated Predictor for Cancer,” Thorax 69, no. 5 (2014): 451–457. 24227199 10.1136/thoraxjnl-2013-203821

[19] R. A. van Boerdonk , T. G. Sutedja , P. J. Snijders , et al., “DNA Copy Number Alterations in Endobronchial Squamous Metaplastic Lesions Predict Lung Cancer,” American Journal of Respiratory and Critical Care 184, no. 8 (2011): 948–956. 10.1164/rccm.201102-0218OC 21799074

[20] C. Pan , B. H. Diplas , X. Chen , et al., “Molecular Profiling of Tumors of the Brainstem by Sequencing of CSF‐Derived Circulating Tumor DNA,” Acta Neuropathologica 137, no. 2 (2019): 297–306. 30460397 10.1007/s00401-018-1936-6 PMC7523750

[21] C. Y. Chiu , “Viral Pathogen Discovery,” Current Opinion in Microbiology 16, no. 4 (2013): 468–478. 23725672 10.1016/j.mib.2013.05.001 PMC5964995

[22] A. Piantadosi , S. S. Mukerji , S. Ye , et al., “Enhanced Virus Detection and Metagenomic Sequencing in Patients With Meningitis and Encephalitis,” MBio 12, no. 4 (2021): e114321. 10.1128/mBio.01143-21 PMC8406231 34465023

[23] P. S. Ramachandran and M. R. Wilson , “Metagenomics for Neurological Infections–Expanding Our Imagination,” Nature Reviews. Neurology 16, no. 10 (2020): 547–556. 32661342 10.1038/s41582-020-0374-y PMC7356134

[24] X. W. Xing , J. T. Zhang , Y. B. Ma , et al., “Metagenomic Next‐Generation Sequencing for Diagnosis of Infectious Encephalitis and Meningitis: A Large, Prospective Case Series of 213 Patients,” Frontiers in Cellular and Infection Microbiology 10 (2020): 88. 32211343 10.3389/fcimb.2020.00088 PMC7066979

[25] W. Gu , A. M. Rauschecker , E. Hsu , et al., “Detection of Neoplasms by Metagenomic Next‐Generation Sequencing of Cerebrospinal Fluid,” JAMA Neurology 78, no. 11 (2021): 1355–1366. 34515766 10.1001/jamaneurol.2021.3088 PMC8438621

