# Supplementary material for: Early Hints of Metagenome Next‐Generation Sequencing and Copy Number Variations Analysis: An Occult Case of Leptomeningeal Metastasis With Rapid Cognitive Decline
Source: Clin Case Rep. 2024 Dec 2;12(12):e9676. doi: 10.1002/ccr3.9676 (PMC11609582; doi:10.1002/ccr3.9676)
Supplement: Supplementary file 1 — Data S1. [file CCR3-12-e9676-s001.doc]

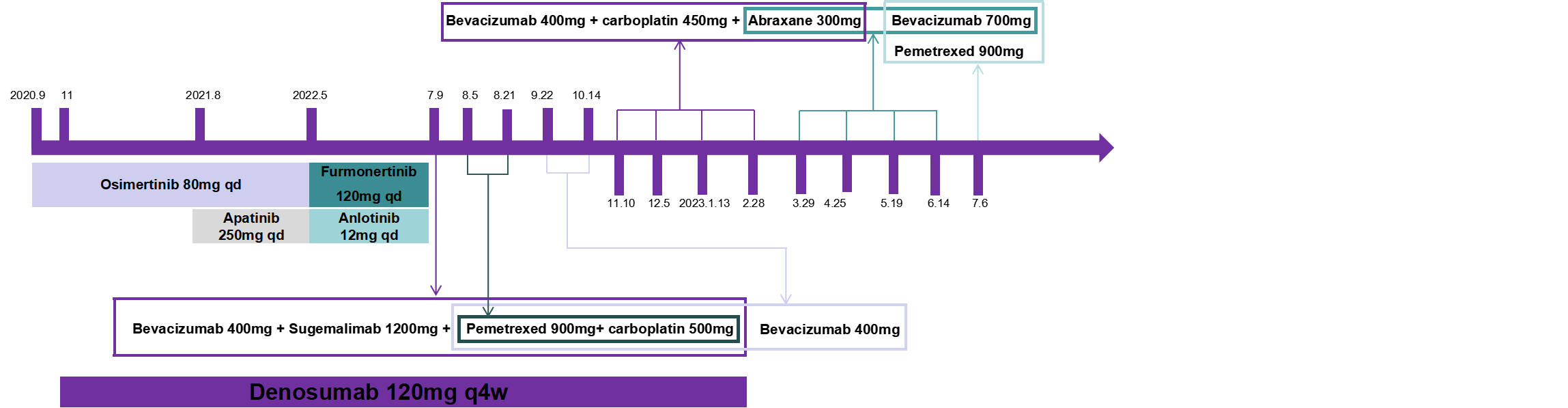


**Supplementary file. 1**

**Supplementary file 1.** Treatment timeline of the patient. Qd: quaque die; q3w: 3 weeks using a; q4w: 4 weeks using a.
